# Comparison of functional and discrete data analysis regimes for Raman spectra

**DOI:** 10.1007/s00216-021-03360-1

**Published:** 2021-05-15

**Authors:** Rola Houhou, Petra Rösch, Jürgen Popp, Thomas Bocklitz

**Affiliations:** 1 Institute of Physical Chemistry, Friedrich Schiller University Jena, Helmholtzweg 4, 07743 Jena, Germany; 2 Department of Photonic Data Science, Leibniz Institute of Photonic Technologies, Member of Leibniz Research Alliance “Leibniz-Health Technologies”, Albert-Einstein-Str. 9, 07745 Jena, Germany

**Keywords:** Raman spectroscopy, Principal component analysis, Functional data analysis, B-splines, Functional principal component analysis

## Abstract

Raman spectral data are best described by mathematical functions; however, due to the spectroscopic measurement setup, only discrete points of these functions are measured. Therefore, we investigated the Raman spectral data for the first time in the functional framework. First, we approximated the Raman spectra by using B-spline basis functions. Afterwards, we applied the functional principal component analysis followed by the linear discriminant analysis (FPCA-LDA) and compared the results with those of the classical principal component analysis followed by the linear discriminant analysis (PCA-LDA). In this context, simulation and experimental Raman spectra were used. In the simulated Raman spectra, normal and abnormal spectra were used for a classification model, where the abnormal spectra were built by shifting one peak position. We showed that the mean sensitivities of the FPCA-LDA method were higher than the mean sensitivities of the PCA-LDA method, especially when the signal-to-noise ratio is low and the shift of the peak position is small. However, for a higher signal-to-noise ratio, both methods performed equally. Additionally, a slight improvement of the mean sensitivity could be shown if the FPCA-LDA method was applied to experimental Raman data.

**Supplementary Information:**

The online version contains supplementary material available at 10.1007/s00216-021-03360-1.

## Introduction

When light interacts with molecules within a sample volume, the result of the interaction is depending on both properties of the sample and the light. The study of such light-matter interactions is done by spectroscopic techniques. In this manuscript, we focus on Raman spectroscopy, which is an inelastic scattering process. The scattered light contains information about energy levels within the molecules, which result from the vibrational and rotational modes. In Raman spectroscopy, the obtained spectral data contain a vast amount of information concerning the molecules within the sample. However, this information cannot directly be used and the extraction of the information needs chemometric methods. Hence, the combination of these chemometric methods with Raman spectroscopy enables the extraction of the relevant information and increases the knowledge regarding the composition of a sample. The combination of Raman spectroscopy and chemometrics has gained popularity and can be used to address a number of tasks, like disease diagnostics and bacteria identification [1–6].

Typically, chemometric methods are split into univariate and multivariate methods, supervised and unsupervised methods, or qualitative and quantitative methods. These methods are typically understood in the discrete case, where the data is discrete. However, a different data analysis approach is needed if functions should be analyzed. Therefore, we can further separate chemometric methods into two additional groups, the discrete group and the functional data analysis group. The chemometric methods in the discrete group, which is also called multivariate data analysis, are most often implemented in Raman spectroscopy. In this group, the key concept is to consider each spectrum as a set of independent points acquired on a specific interval, e.g., wavenumber or frequency. Moreover, various models and tasks are contained in this group, e.g., dimension reduction, clustering, regression, and classification [7, 8]. For instance, the principal component analysis (PCA) is a well-known method to reduce the dimensionality in spectral data analysis [9–11]. For classification tasks, many methods are developed and implemented for spectroscopic measurements, e.g., linear discriminant analysis (LDA) [12–14], support vector machine [15–17], and neural networks [18]. In contrast to the methods in the discrete data analysis group, a spectrum is modeled as a function or a curve in the functional data analysis group. These functions are latent and infinite-dimensional, which cannot be calculated analytically and they need to be approximated. The study of these functions falls under the name functional data analysis (FDA), which originally was introduced by Ramsay et al. [19, 20]. It involves smoothing technique, data reduction, adjustment for clustering, functional linear modeling, and forecasting methods [21–24]. Initially, Ramsay et al. developed the FDA to analyze, model, and predict time series and then expanded the FDA to cover other data types. The advantages of using chemometric methods in the functional group are that FDA overcomes the curse of dimensionality. Moreover, the assumption that adjacent observations should be independent is not needed in the functional data analysis group, while in the discrete group, this assumption is needed but often violated.

The chemometric methods used in the functional data analysis group are adapted from the discrete ones. For instance, the functional principal component analysis (FPCA) is an adapted version of a discrete PCA. The FPCA deals with data in the form of functions and was developed by Dauxois et al. [25]. James et al. [26] introduced the functional linear discriminant analysis (FLDA) extended from the classical LDA method, where the predictor variables are curves or functions. Mas et al. [27] introduced the functional version of linear regression for random functions by considering the first-order derivative. Furthermore, the application of these functional methods was spread to various fields, including medicine [28–30], economics [31, 32], agriculture [33], linguistics [34–36], and behavior sciences [37]. However, as far as the authors know, they were never applied to Raman spectral data, and their application was limited to mass spectrometry [38] and near-infrared spectroscopy [39]. Although the Raman spectral data are in nature functions of wavenumber or frequency, they are acquired discretely on finite points due to the used spectroscopic setup. In this manuscript, we evaluated the analysis of Raman spectra in the functional framework for the first time. This evaluation was achieved by comparing the performance of the functional principal component analysis followed by the linear discriminant analysis (FPCA-LDA) to the classical principal component analysis followed by the linear discriminant analysis (PCA-LDA) on simulation and experimental Raman spectral data.

The manuscript is divided into four sections. First, we presented the theoretical background of the functional data analysis and the functional principal component analysis. The workflow of the two methods, the classical PCA-LDA and the FPCA-LDA, is explained in the “Material and method” section. In the “Results” section, the comparison between these two methods applied on simulation and experimental Raman data is shown. Finally, we summarized the main findings in the “Conclusion” section.

## Theoretical background

Functional data analysis (FDA) refers to the analysis of data in the form of functions. For instance, if our studied data is collected in a matrix ***x*** ∈ *ℝ*^*N* × *p*^, then each row of this data is considered a function. The underlying idea of FDA is that we assume the existence of some functions ***x***_***i***_(*t*), *i* = 1, …, *N*, giving rise to the observed data. Therefore, this function is treated as one entity instead of a sequence of individual measured variables.

### Functional approximation

The analysis of functions in the functional framework is done in some functional space, which is often assumed to be a Hilbert space, such as *L*^2^(*I*) defined on a compact interval *I*. However, we cannot analytically calculate these functions from the data, and we need to apply basis functions to approximate them. These basis functions represent a set of available functions *φ*_*k*_, *k* = 1, 2, …, *K* that are mathematically independent of each other. With a linear combination of adequately large number of these basis functions, we can approximate our observed data. This approximation can be formulated by a linear expansion of *K* known basis functions *φ*_*k*_ as follows:

1 $$ {\boldsymbol{x}}_{\boldsymbol{i}}(t)=\sum \limits_{k=1}^K{c}_k{\varphi}_k(t). $$

Likewise Eq. (1) can be expressed in matrix notation, as follows [40, 41]:

2 $$ {\boldsymbol{x}}_{\boldsymbol{i}}={\boldsymbol{C}}^{\prime}\boldsymbol{\phi} ={\boldsymbol{\phi}}^{\prime}\boldsymbol{C}, $$

where ***C*** is the vector of length *K* of the coefficients *c*_*k*_ and ***ϕ*** is the functional vector whose elements are the basis functions *φ*_*k*_. The choice of the basis functions depends on the studied problem, where they should have features similar to those known to belong to the estimated functional data. However, most functional data analysis involves either a Fourier basis for periodic data or a B-spline basis for non-periodic data. The B-spline basis functions are the most common choice for approximating spectral data. These basis functions are defined on a specific interval *I* with an order *O* and a knot vector. First, the interval *I* is divided into subintervals where the corresponding endpoints are represented by the knot vector {*t*_1_, …, *t*_*K* + *O*_}. After imposing continuity and smoothness conditions, the *K* B-spline basis functions of order *O* can be defined using Eq. (3):

3 $$ {\displaystyle \begin{array}{c}{\varphi}_{k,1}(t)=\left\{\begin{array}{c}1,\kern0.5em {t}_k\le t<{t}_{k+1}\\ {}0,\kern0.5em else\end{array}\right.\\ {}{\varphi}_{k,O}(t)=\frac{t-{t}_k}{t_{k+O}-{t}_k}{\varphi}_{k,O-1}(t)+\frac{t_{k+O+1}-t}{t_{k+O+1}-{t}_{k+1}}{\varphi}_{k+1,O-1}(t).\end{array}} $$

Consequently, the approximation is achieved by calculating the coefficients *c*_*k*_. Various methods can be implemented for this purpose; however, the simplest one is to minimize the sum of squared errors or the so-called least squares estimation using Eq. (4):

4 $$ \mathrm{SMSSE}\left({\boldsymbol{x}}_{\boldsymbol{i}}\right)=\sum \limits_{j=1}^p{\left|{x}_{ij}-\sum \limits_{k=1}^K{c}_k{\varphi}_k\left({t}_j\right)\right|}^2={\left\Vert {\boldsymbol{x}}_{\boldsymbol{i}}-\boldsymbol{\phi} \boldsymbol{C}\right\Vert}^2. $$

The choice of the number of basis functions *K* has an influence on the approximation and it should be chosen carefully. With a high number of basis function, we risk fitting noise, and with a small *K*, we might remove some important aspect of the functional data. Therefore, methods exist, which can be used to obtain an optimal number of basis functions, e.g., the elbow method, the bias/variance trade-off method, and the stepwise variable selection method [42].

In contrast to the multivariate data analysis, the functional data in the FDA procedure are inherently infinite-dimensional, which makes the computation at any value *t* possible. Besides, the underlying functions in FDA are smooth, but the observed data are often not due to the presence of noise in the measurements. Therefore, a higher level of variation in the observed data can make the extraction of a stable estimate of the functional data challenging. Furthermore, sparse and irregular observed longitudinal data can be analyzed in FDA.

### Functional principal components analysis (FPCA)

Functional principal component analysis (FPCA) is a key dimension reduction tool for functional data, and it is considered the most popular method in the functional analysis. Similarly to the classical principal component analysis (PCA), we need to examine the variance-covariance matrix/function in order to calculate the principal components. The theoretical background of FPCA [43] is shown below in detail; however, since it represents a functional version of the classical PCA, a short introduction to the theory of PCA is first presented.

The key idea of PCA is to construct the *i*^th^ principal component ***f***_***i***_ as a linear combination of the variable ***x***_***i***_ = (*x*_*i*1_, …, *x*_*ip*_)^′^ as shown in Eq. (5):

5 $$ {\boldsymbol{f}}_{\boldsymbol{i}}=\sum \limits_{j=1}^p{\beta}_j{x}_{ij},i=1,\dots, N $$

where *β*_*j*_ is the weight coefficient of the observed values *x*_*ij*_ of the *j*^th^ variable. In PCA, we start by finding the weight vector ***β***_**1**_ = (*β*_11_, …, *β*_*p*1_)^′^ such as ${f}_{i1}={\sum}_j{\beta}_{j1}{x}_{ij}={\beta}_1^{\prime }{x}_i$ have the largest possible mean square ${N}^{-1}{\sum}_i{f}_{i1}^2$ subject to the following constraint ${\sum}_j{\beta}_{j1}^2={\left\Vert {\boldsymbol{\beta}}_{\mathbf{1}}\right\Vert}^2=1$. Then, we proceed to the second step and subsequent steps until reaching a desired number, which should be less or equal to the number of variables *p*. On the *m*^th^ step, similar to the first step, we compute a new weight vector ***β***_***m***_ and new values *f*_*im*_; thus, the values *f*_*im*_ have maximum mean square, subject to the constraint ‖***β***_***m***_‖^2^ = 1 and the *m* − 1 additional constraints ${\sum}_j{\beta}_{jq}{\beta}_{jm}={\boldsymbol{\beta}}_{\boldsymbol{q}}^{\prime }{\boldsymbol{\beta}}_{\boldsymbol{m}}=0,q<m$. These *f*_*im*_ are called the principal component scores.

In contrast to the classical PCA, the variable values in FPCA are function ***x***_***i***_(*t*), and the equivalent notation of ***β*** and ***x*** in FPCA are the functions ***β***(*t*) and ***x***(*t*). Therefore, the principal component scores corresponding to the weight function are illustrated in Eq. (6):

6 $$ {\boldsymbol{f}}_i=\int \boldsymbol{\beta} {\boldsymbol{x}}_{\boldsymbol{i}}=\int \boldsymbol{\beta} (t)\boldsymbol{x}(t) dt. $$

The first step in FPCA is to find the weight function ***β***_**1**_(*s*) in such a way that it maximizes ${N}^{-1}{\sum}_i{f}_i={N}^{-1}{\sum}_i{\left(\int {\beta}_1{x}_i\right)}^2$ and subject to the unit sum of squares constraint ∫***β***_**1**_(*s*)^2^ = 1. Then, we proceed to the *m* step, where we find the weight function *β*_*m*_ that satisfies the orthogonality constraints ∫***β***_***q***_***β***_***m***_ = 0, *q* < *m*. However, in most principal component analysis applications, finding the principal components is equivalent to finding the eigenvalues and eigenfunctions of the covariance function. Therefore, the covariance function ***v***(***s***, ***t***) is defined as follows:

7 $$ \boldsymbol{v}\left(\boldsymbol{t},\boldsymbol{s}\right)={N}^{-1}\sum \limits_{i=1}^N{\boldsymbol{x}}_{\boldsymbol{i}}(t){\boldsymbol{x}}_{\boldsymbol{i}}(s) $$

And each eigenfunction ***β***_***j***_(*t*) for an appropriate eigenvalue *ρ* satisfies

8 $$ \int \boldsymbol{v}\left(\boldsymbol{t},\boldsymbol{s}\right)\boldsymbol{\beta} (s) ds=\rho \boldsymbol{\beta} (t) $$

The left side of this equation is an integral transform ***V*** of the weight function ***β*** that can be defined by Eq. (9), and it is called the covariance operator ***V***. Therefore, we may also express the eigenequation directly as Eq. (10), where ***β*** is an eigenfunction rather than an eigenvector.

9 $$ \boldsymbol{V}\boldsymbol{\beta } =\int \boldsymbol{v}\left(.,\boldsymbol{t}\right)\boldsymbol{\beta} (t) dt $$

10 $$ \boldsymbol{V}\boldsymbol{\beta } =\rho \boldsymbol{\beta} $$

In classical PCA, the number of variables is equal to *p*. In contrast, in the case of functional PCA (FPCA), the number of variables refers to the number of function values which is infinity. However, given that the functions ***x***_***i***_(*t*) are not linearly dependent, the operator will have rank *N* − 1, and there will be *N* − 1 nonzero eigenvalues.

## Material and method

Our study is divided into two parts. First, we applied the classical principal component analysis as described previously on our data stored in a *N* × *p* matrix ***x***, and then we extracted the principal component scores matrix ***β***. Instead of applying the linear discriminant analysis directly on the data, we used these scores as input to the LDA model. This latter model calculates *L* − 1 linear discriminant functions to separate *L* groups, as shown in Eq. (11):

11 $$ \boldsymbol{LD}=\boldsymbol{\Omega} \boldsymbol{\upbeta} +{\boldsymbol{b}}_{\mathbf{0}} $$

which represents a linear combination of the principal component scores, where **Ω** represents the weight matrix and ***b***_**0**_ is the bias. Briefly, these functions are calculated by maximizing the between-group variance illustrated in Eq. (12):

12 $$ \boldsymbol{B}=\sum \limits_{l=1}^L{n}_l\left({\overline{\boldsymbol{\beta}}}_{\boldsymbol{l}}-\overline{\boldsymbol{\beta}}\right){\left({\overline{\boldsymbol{\beta}}}_{\boldsymbol{l}}-\overline{\boldsymbol{\beta}}\right)}^{\prime } $$

and minimizing the within-group variation calculated in Eq. (13):

13 $$ \boldsymbol{W}=\sum \limits_{l=1}^L\left({\boldsymbol{\beta}}_{.,\boldsymbol{l}}-{\overline{\boldsymbol{\beta}}}_{\boldsymbol{l}}\right){\left({\boldsymbol{\beta}}_{.,\boldsymbol{l}}-{\overline{\boldsymbol{\beta}}}_{\boldsymbol{l}}\right)}^{\prime }. $$

**Fig. 1:**
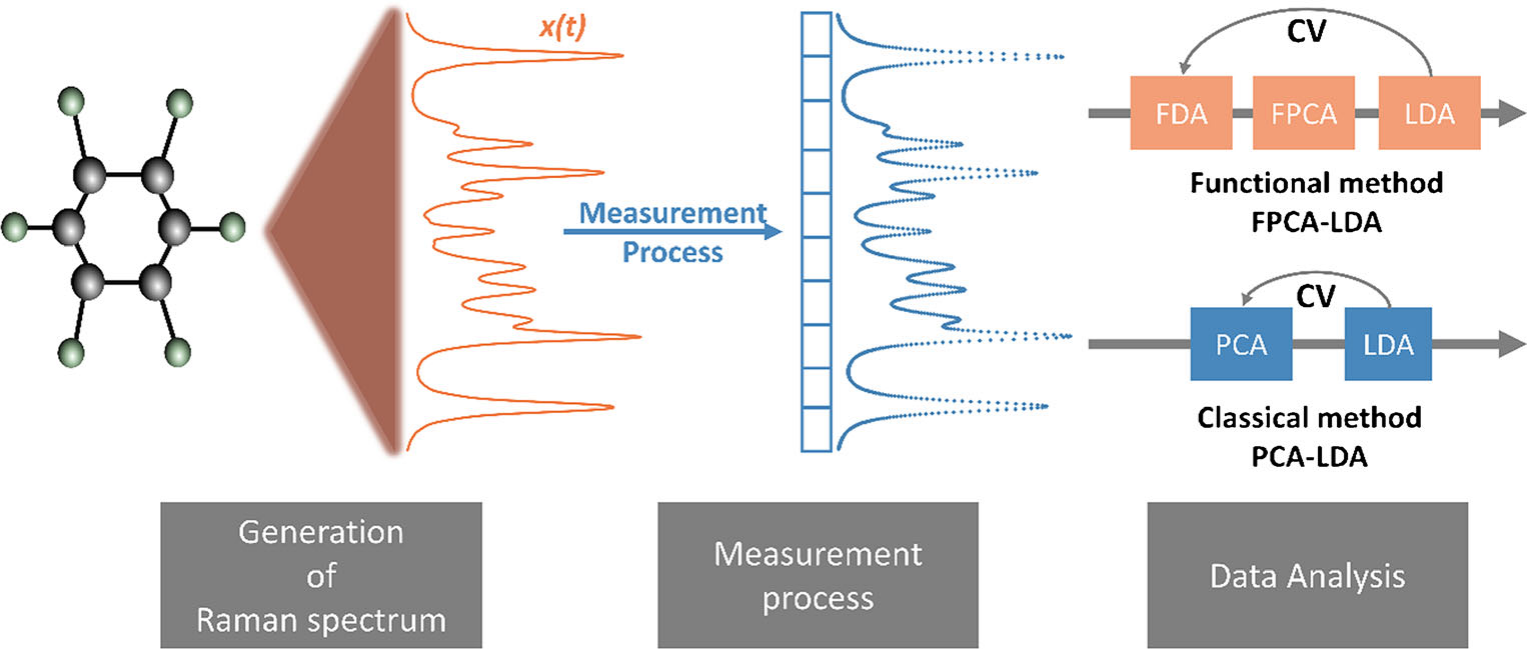
The motivation and the workflow of the PCA-LDA and the FPCA-LDA methods. On the left, the generation of a Raman spectrum is visualized, which yields a Raman spectrum in functional form. However, due to the measurement process (in the middle), we acquire the spectrum in a discrete manner. Due to the used multichannel detector, a measured Raman spectrum is characterized by a vector of intensities. On the right, the data analysis workflow that aims to compare the classical principal component analysis followed by linear discriminant analysis (PCA-LDA) and the functional principal component analysis followed by linear discriminant analysis (FPCA-LDA) is shown. Both methods include a cross-validation (CV) loop and the functional data is constructed by using B-splines

In contrast to the discrete framework, we implemented the functional framework by the following steps. We transformed our discrete data into functions using cubic B-spline basis functions where the order *O* is equal to 4 and we approximated the coefficients *c*_*k*_ using Eq. (1) and the least square method. Then, the functional principal component analysis was applied to these functional versions of our data, and the functional scores were extracted. We then apply LDA on these functional scores. The testing sets in the classical PCA-LDA and FPCA-LDA were projected into the corresponding principal component space. Moreover, both methods were used inside a cross-validation loop and the results are obtained using group scripts developed in Gnu R software. The motivation and the workflow of both PCA-LDA and FPCA-LDA are illustrated in Fig. 1.

## Results

We tested the performance of the functional data analysis by comparing the functional principal component analysis followed by linear discriminant analysis (FPCA-LDA) and the classical principal component analysis followed by linear discriminant analysis (PCA-LDA) on both simulation and experimental Raman data.

### Simulation data

First, we simulated Raman spectra of two classes (a normal group and an abnormal group) with three peaks. The abnormal group was generated by slightly shifting one of the peaks from the peak position used in the normal group. This situation is often occurring in biomedical Raman spectroscopy, when for example the protein’s secondary structure changes between two groups or if an isotope labeling was applied. We did that in two scenarios without adding a background and with adding a background contribution. The two classes in the simulated Raman spectra are referred to as normal and abnormal spectra. The number of pixels in each spectrum is equal to 1024. The abnormal spectra were constructed similarly to the normal spectra with three peaks, but only one of these peaks was shifted by one of the following values$\left(\Delta \overset{\sim }{\upsilon}\right)$: 0.001, 0.003, 0.005, 0.01, 0.02, 0.025, 0.05. The total number of spectra in each class was set to 100 spectra. In addition, the simulation data was constructed for different signal-to-noise ratio (SNR) cases: 0.5, 1, 2, 3, 5, 10, 30, 50, 100. We end up with 63 cases of simulation data where each of the datasets includes 200 spectra. The same cases were constructed including a random background. The parameters of the simulation are summarized in Supplementary Information (ESM) Table S1. An example of the mean spectra for the simulated Raman where the shift in the peak position is equal to 0.05 and the SNR is 30 is shown in Fig. S1 in the ESM.

**Fig. 2:**
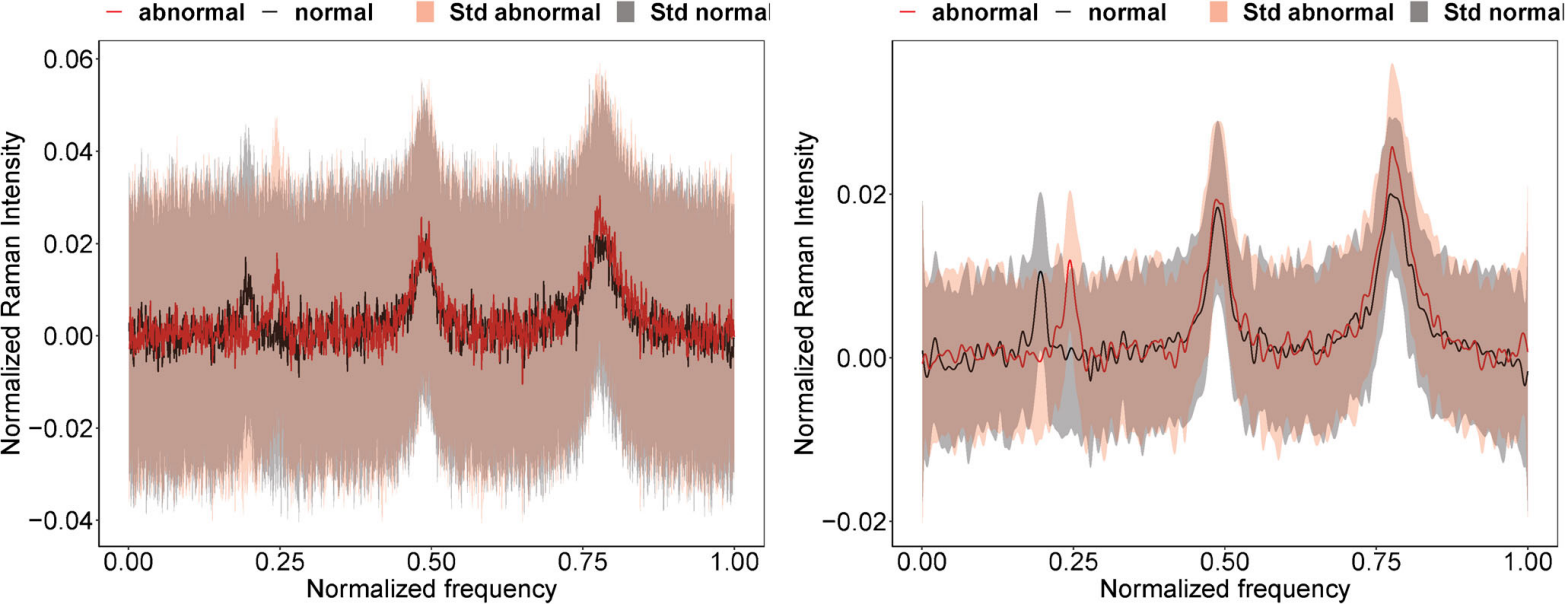
The mean spectra per class for the simulation without a background in the case where $\varDelta \overset{\sim }{\upsilon }=0.05$ and SNR = 0.5. The left plot represents the discrete simulated Raman mean spectra per each class, while the right plot represents the functional version of these simulated Raman spectra using 190 basis functions

For the simulated Raman spectra without background, 190 B-splines are used in the FDA approximation. An illustration of the first 10 B-splines basis functions can be found in the ESM in Fig. S2. The choice of the number of basis functions is calculated based on the elbow method. We first calculated the root-mean-square error, and the optimal point is chosen, which refers to the largest distance to the line that joins the first and last values. After choosing the number of basis functions, we transform our discrete spectra into functional version using Eq. (1). The mean spectra for both discrete and functional versions in the case where $\Delta \overset{\sim }{\upsilon }=0.05$ and SNR = 0.5 are illustrated in Fig. 2. On the left, the original mean spectra for the normal and the abnormal classes are shown in black and red, respectively. In comparison, the functional mean spectra for normal and abnormal classes are shown on the right in black and red, respectively. The functional spectra represent well the original spectra, where the shape of the peaks is preserved and a significant reduction of the noise was observed.

The results of the functional approximation for all the constructed cases are illustrated in Figs. S3 and S4 in the ESM. In ESM Fig. S3, we showed the mean spectra for the simulated Raman without background per shift when the SNR is equal to 0.5 for the original discrete spectra and its functional approximation. On the left, the original mean spectra for the simulation without background are shown. The black plot represents the mean spectra of the normal class, while the colored plots refer to the mean spectra of the abnormal class corresponding to the specific shift values $\Delta \overset{\sim }{\upsilon }$. On the right, the mean spectra of the functional approximation of the corresponding discrete spectra are illustrated. When comparing the functional mean spectra with the discrete mean spectra, we can deduce a significant reduction of noise and an improvement in the peak shape estimation.

In ESM Fig. S4, we showed one spectrum per class for each SNR and for a specific peak shift $\Delta \overset{\sim }{\upsilon }=0.01$. On the left, the original spectra are illustrated. In each row, the normal and abnormal spectra are plotted for all SNR values. On the right, the functional approximations for each class are illustrated for the nine cases of SNR. In all these cases, we used 190 B-spline basis functions in the functional approximation. For a SNR which is larger than 5, the functional approximations perfectly represent the original spectra with almost noiseless reconstruction. Although the functional approximation also fit noise for the lower SNR values due to a high number of basis used, a significant reduction of noise is noticed in the approximation, and it maintains potentially the peak shape in the case of lower SNR values.

**Fig. 3:**
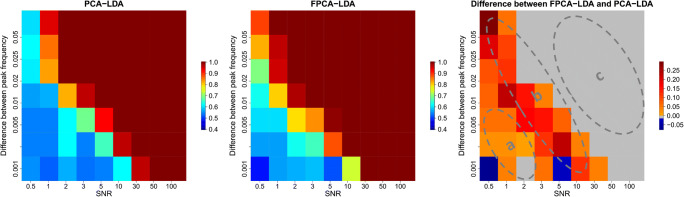
The mean sensitivities of applying PCA-LDA and FPCA-LDA on the simulated Raman data without background and the difference between both methods. The mean sensitivity of PCA-LDA, the mean sensitivity of FPCA-LDA, and the difference between the mean sensitivities of FPCA-LDA and PCA-LDA is illustrated on the left, middle, and right, respectively. In region c, both FPCA-LDA and PCA-LDA perform equally. While in regions a and b, the FPCA-LDA method performs better compared to PCA-LDA

The two approaches (PCA-LDA and FPCA-LDA) explained previously were applied to the simulated Raman spectra that contain no background. For both methods, we used 10-fold cross-validation, and the number of components chosen was 50 components. The mean sensitivity was used as an evaluation metric, and it is illustrated in a heat map in Fig. 3. In region c of Fig. 3, PCA-LDA and FPCA-LDA perform perfectly with 100% mean sensitivity, due to the clear distinction between the normal and the abnormal classes. While in regions a and b of the same figure, the FPCA-LDA performs better in most of the cases, due to the fact that in the functional approximation, the obtained functions contain less noise and an improvement of the shape of the peaks in these functions was also detected. However, in few cases, the PCA-LDA provides better mean sensitivity. This result might refer to the choice of the number of basis functions that should not be too large that the approximation includes more noise or too small that the approximation excludes some relevant features in the approximation.

In the simulated Raman spectra that include a random background, 180 B-spline basis functions are used in the FDA approximation. The choice of the number of basis functions is calculated similarly to the simulation without background based on the elbow method. After choosing the number of basis functions, we transform our discrete spectra into functional versions using Eq. (1). The mean spectra of both discrete and functional versions in the case $\Delta \overset{\sim }{\upsilon }=0.05$ and SNR = 0.5 are illustrated in the ESM in ESM Fig. S5. On the left, the original mean spectra for the normal and the abnormal classes are shown in black and red, respectively. The functional mean spectra for normal and abnormal classes are shown on the right in black and red, respectively. The functional spectra represent well the original spectra, where the shape of peaks is preserved, and a significant reduction of noise was observed.

The results of the functional approximation are illustrated in the ESM in Figs. S6 and S7. In ESM Fig. S6, we showed the mean spectra for the simulated Raman data with background per each shift and for SNR 0.5 for the original discrete spectra and its functional approximation. On the left, the original mean spectra for the simulation with a random background are illustrated. The black plot represents the mean spectrum of the normal class, while the colored plots refer to the mean spectra of the abnormal class corresponding to the specific shift values $\Delta \overset{\sim }{\upupsilon}$. On the right of ESM Fig. S6, the mean spectra of the functional approximation of the corresponding discrete spectra are shown. When comparing the functional mean spectra with the discrete mean spectra, we can deduce a significant reduction of the noise and an improvement in the peak shape estimation.

In ESM Fig. S7, we showed for a specific peak shift position $\Delta \overset{\sim }{\upupsilon}=0.01$, one spectrum per class for each SNR case. On the left, the original spectra are illustrated. In each row, the normal and abnormal spectra are plotted for all SNR values. On the right, the functional approximations for each class are illustrated for the nine cases of SNR. In all these cases, we used 180 B-spline basis functions in the functional approximation. For a SNR which is larger than 5, the functional approximations perfectly represent the original spectra with almost noiseless construction. Although the functional approximation also fit noise for the lower SNR values due to a high number of basis used, a significant reduction of noise is observed in the approximation, and these functions preserved the peak shape in the case of lower SNR values.

**Fig. 4:**
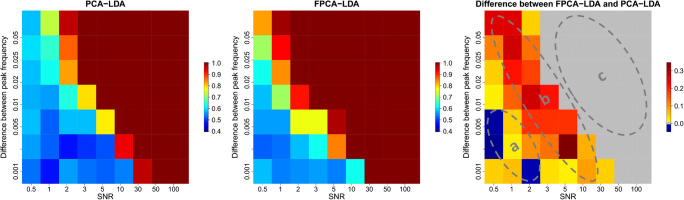
The mean sensitivity of applying PCA-LDA and FPCA-LDA on the simulated Raman data with background and the difference between both methods. The mean sensitivity of PCA-LDA, the mean sensitivity of FPCA-LDA, and the difference of the mean sensitivities are illustrated on the left, middle, and right, respectively. In region c, both FPCA-LDA and PCA-LDA perform equally. While in regions a and b, the FPCA-LDA method performs better compared to PCA-LDA

The two approaches (PCA-LDA and FPCA-LDA) explained previously were applied on this simulated Raman spectra that contain a random background. For both methods, we used 10-fold cross-validation, and the number of components chosen was 50 components. The mean sensitivity was used as an evaluation metric, and the results are illustrated in a heat map in Fig. 4. In region c of Fig. 4, PCA-LDA and FPCA-LDA perform perfectly with 100% mean sensitivity, due to the clear distinction between the normal and the abnormal class. While in regions a and b of the same figure, in most cases, the FPCA-LDA performs better, due to the fact that in the functional approximation, less noise and an improvement of the shape of the peaks exist. However, in some cases, the PCA-LDA provides a better mean sensitivity. This result might refer to the choice of the number of basis functions that should not be too large and too small. In order to test the significance of these findings, the non-parametric Kruskal-Wallis test was implemented. We tested the significance of the difference of the classification performance of both methods (FPCA-LDA and PCA-LDA). We performed this test for the simulation without and with background. All corresponding *p*-values are shown in ESM Fig. S10 where values less than 0.05 and values larger than 0.05 are highlighted in blue and in red, respectively.

In conclusion, we showed that FPCA-LDA and PCA-LDA perform equally in region c for both simulations (with and without background). However, FPCA-LDA performs better in both regions a and b of Fig. 3 and Fig. 4 for the simulation with and without background, respectively. The difference of performance is only significant in region b, which was determined by a Kruskal-Wallis test (ESM Fig. S10). This test showed that the performance difference of FPCA-LDA and PCA-LDA is only significant in region b for both simulations with and without background.

### Experimental data

The experimental data was published elsewhere [44]. Shortly, a Raman microscope with an excitation wavelength of 532 nm was used for the acquisition of the experimental spectra. The naphthalene-degrading soil bacteria *Rhodococcus opacus* DSM 8531 (*R. opacus*), *Novosphingobium aromaticivorans* DSM 12444 (*N. aromaticivorans*), and *Cupriavidus basilensis* DSM 9750 (*C. basilensis*) were included in the study and purchased from the Leibniz Institute DSMZ-German Collection of Microorganisms and Cell Cultures. Each Raman spectrum is a spectrum acquired from a single cell of around 75 to 100 single cells. Throughout the experiments, three batches were cultivated and measured. The aforementioned microorganisms are cultivated separately in water and in heavy water (D_2_O). Through this fact, hydrogen atoms are exchanged by deuterium atoms and the C-H bond is exchanged by a C-D bond. The corresponding stretching vibration band (C-D stretching) is shifted in the Raman silent region (for more details on the experimental setup and pre-processing, we refer to Kumar et al. [44]). In the following, the two methods, PCA-LDA and FPCA-LDA, were applied on both raw and pre-processed experimental data. The total number of spectra used is 2262 and 1131 spectra for the raw experimental and pre-processed dataset, respectively. The pre-processing consists of a combination of two spectra for spike correction so the preprocessed dataset features less spectra than the raw dataset. We applied two approaches for two cross-validation, namely the leave one batch out cross-validation (LOBOCV) and the 10-fold cross-validation (10-fold CV).

**Fig. 5:**
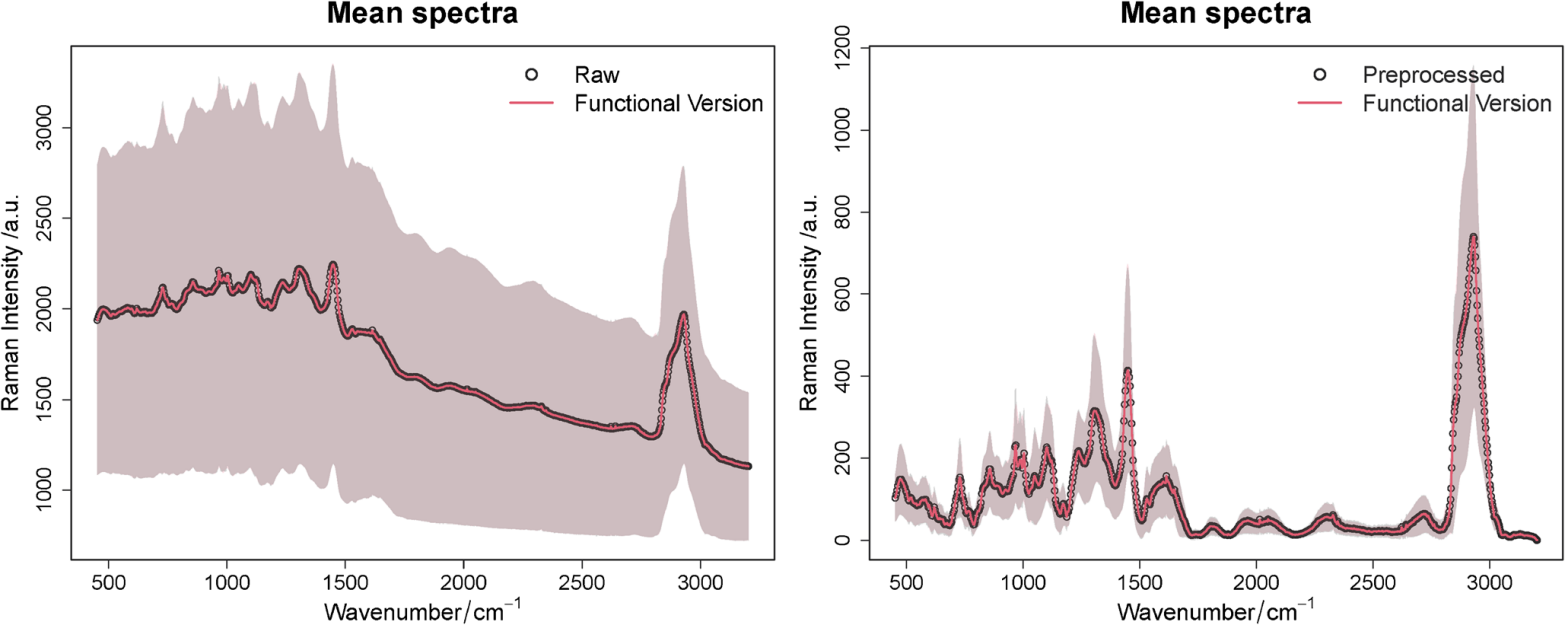
The discrete and the functional mean spectra for the raw and pre-processed Raman data. On the left, the raw Raman data is illustrated through their discrete mean spectra and their functional mean spectra in black and red, respectively. In the right panel, the pre-processed Raman data is also illustrated through their discrete mean spectrum and their functional mean spectrum in black and red, respectively

The first step in the FPCA-LDA is to approximate our experimental Raman data into functions. Therefore, the B-spline basis functions are used for both the raw and pre-processed experimental Raman data. The number of basis functions implemented is equal to 200. The mean spectra for both raw and pre-processed Raman data and their functional mean spectra are shown in Fig. 5. The functional mean spectra (SNR = 278.25) for the raw experimental data represent well the original data (SNR = 272.38) with a slight reduction of noise as shown in the left panel of Fig. 5. In the right side of this figure, the functional mean spectra (SNR = 307.14) of the pre-processed experimental Raman data (SNR = 302) are illustrated. Like the raw functional mean, the pre-processed functional mean represents well the original data and a slight reduction of noise is observed. The elbow method suggested that the optimal number of basis functions is equal to 80 basis functions. However, we increased this number to 200 basis functions because we wanted the functional approximation to include the C-D/C-H region with high quality.

**Fig. 6:**
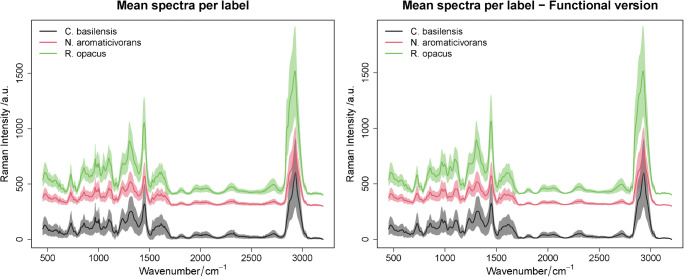
The mean spectra per class (*C*. *basilensis*, *N*. *aromaticivorans*, *R*. *opacus*) for the pre-processed experimental data (left) and their functional approximation (right). The discrete mean spectra per class are shown on the left. Their functional counter parts are illustrated on the right side. A reduction of noise in the functional version is deduced, where the mean intensity with the standard deviation for specific wavenumbers of both versions is illustrated in ESM Table S2. Beside this noise reduction, no Raman spectral features are removed

The functional data analysis was applied to the pre-processed experimental data for each of the classes mentioned above, and the discrete mean spectra and their functional version are shown in Fig. 6. A slight reduction of noise in the functional approximation is visible (see ESM Table S2), and this refers to the fact that a large number of basis functions are used and that the SNR of the pre-processed Raman data is high (approximately equal to SNR = 302). This SNR is approximated by calculating the ratio of the peak amplitude at the C-H band (2930 cm^−1^) to the standard deviation of the region between 2408 and 2578 cm^−1^. The functional approximation showed an improved SNR which is equal to 307.14.

**Fig. 7:**
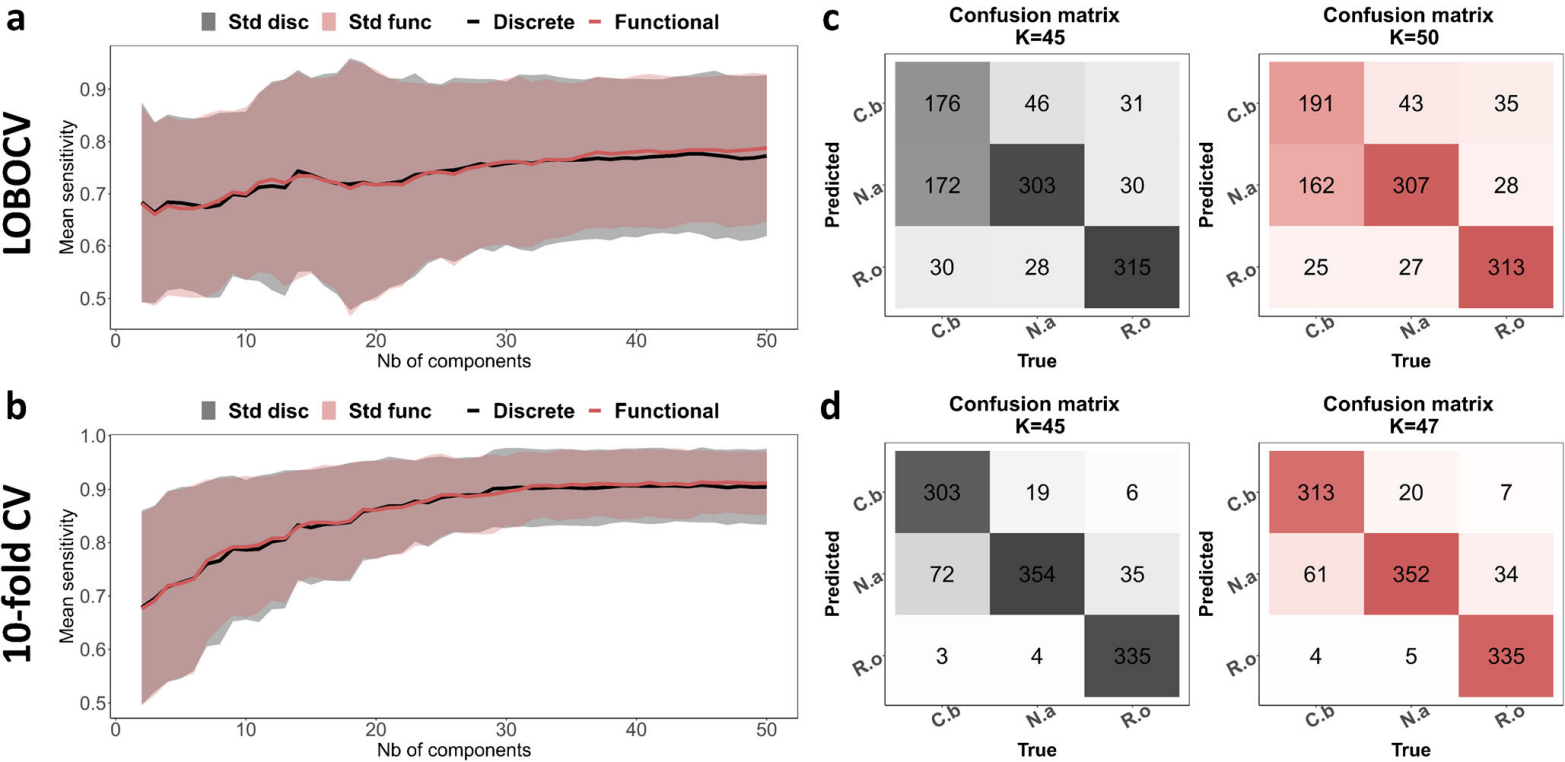
The mean sensitivities and the confusion matrices of PCA-LDA and FPCA-LDA methods using LOBOCV and 10-fold CV using the pre-processed Raman data. Panel **a** represents the mean sensitivities of PCA-LDA and FPCA-LDA methods using LOBOCV in black and red, respectively. The confusion matrices of the models with the highest mean sensitivity are illustrated in panel **c**. Panel **b** refers to the mean sensitivities of the PCA-LDA and the FPCA-LDA methods using 10-fold CV in black and red, respectively. The confusion matrices of the models with highest mean sensitivity are illustrated in panel **d**

We aim to compare both approaches that were explained in the workflow in Fig. 1. Therefore, we implemented the PCA-LDA method on the discrete pre-processed spectra and the FPCA-LDA method on the functional approximation of these spectra. In both methods, we tested the model using the two cross-validation schemes (LOBOCV and 10-fold CV), and the results are summarized in Fig. 7.

The comparison between the PCA-LDA and FPCA-LDA methods using the LOBOCV is illustrated in the first row of Fig. 7. The mean sensitivities are shown in panel a. Similar performance for both methods can be shown with a slight improvement in the values for the FPCA-LDA method, in particular in the region where the number of components is larger than 32. A reduction of the standard deviation is visible in the case of the FPCA-LDA method. Although the functional data analysis resulted in smoothed function, this did not affect the classification output. Therefore, the functional approach conserved the important features needed in the classification. The maximum mean sensitivity for the PCA-LDA method refers to the model with 45 components with a mean sensitivity of 0.77 ± 0.16. However, the maximum sensitivity for the FPCA-LDA method refers to a model with 50 components with a value of 0.79 ± 0.14. The corresponding confusion matrices for the model with the highest mean sensitivities are illustrated in panel c in black and red for PCA-LDA and FPCA-LDA, respectively. In the second row of Fig. 7, the mean sensitivities of PCA-LDA and FPCA-LDA using the 10-fold CV are shown. Similarly to panel a, both methods are performing analogously. A small reduction of the standard deviation is shown in the case of the FPCA-LDA. Even though functional data analysis resulted in smoothed function, this did not affect the classification output. Therefore, the functional approach conserved the important features needed for the classification. The maximum mean sensitivity for the PCA-LDA method refers to the model that includes 45 components with a value of 0.91 ± 0.07. However, the maximum sensitivity for the FPCA-LDA method refers to a model with 47 components with a value of 0.91 ± 0.06. The corresponding confusion matrices for the model with the highest mean sensitivities are illustrated in panel d for PCA-LDA and FPCA-LDA methods in black and red, respectively.

The functional data analysis was also applied to the raw experimental data, and the discrete mean spectra and their functional versions are shown in the ESM in Fig. S8. In these plots, a slight reduction of noise in the functional approximation is visible. This small noise reduction refers to the fact that a large number of basis functions are used and that our experimental Raman data contain less noise (SNR = 272.38). Also in the case of the raw data, we want to compare both approaches that were explained in the workflow in Fig. 1. Therefore, we implemented the PCA-LDA method on the discrete raw spectra and the FPCA-LDA method on the functional approximation of these spectra. In both methods, we tested the model with two cross-validation schemes (LOBOCV and 10-fold CV), and the results are summarized in the ESM in Fig. S9.

The comparison between the PCA-LDA and FPCA-LDA using the LOBOCV is illustrated in the first row of ESM Fig. S9. The mean sensitivities are shown in panel a. Similar performance can be shown for both methods with a slight improvement for the FPCA-LDA method. Particularly in the region where the number of components is larger than 40 and in the region where the number of components is around 20 components, the FPCA-LDA performs better. Additionally, a reduction of the standard deviation is visible in the case of the FPCA-LDA. Although functional data analysis resulted in a smoothed function, this did not affect the classification output. Therefore, the functional approach conserved the important features needed in the classification. The maximum mean sensitivity for the PCA-LDA method was achieved by a model that includes 48 components and the mean sensitivity was 0.77 ± 0.16. However, the maximum sensitivity for the FPCA-LDA method was seen for a model with 44 components and the mean sensitivity was 0.78 ± 0.15. The corresponding confusion matrices for the model with the highest mean sensitivities are illustrated in panel c in black and red for PCA-LDA and FPCA-LDA methods, respectively. In the second row of ESM Fig. S9, the mean sensitivities of PCA-LDA and FPCA-LDA methods using the 10-fold CV are visible. Similar to panel a, both methods perform analogously with a slight improvement in the case where the FPCA-LDA method was applied, particularly in the region where the number of components is larger than 20 components. Furthermore, a reduction of the standard deviation is shown in the case of the FPCA-LDA method. Although functional data analysis resulted in smoothed function, this did not affect the classification output. Therefore, the functional approach conserved the important features needed for the classification. The maximum mean sensitivity for the PCA-LDA method refers to the model that includes 50 components with a value of 0.89 ± 0.08. However, the maximum sensitivity for the FPCA-LDA method could be seen for a model with 50 components (0.9 ± 0.06). The corresponding confusion matrices for the models with highest mean sensitivities are illustrated in panel d in black and red for the PCA-LDA and the FPCA-LDA methods, respectively.

## Conclusion

In this manuscript, we tested the application of the functional data analysis on Raman spectral data. Therefore, we compared the functional approach, e.g., the functional principal component analysis followed by a linear discriminant analysis (FPCA-LDA) to the classical approach, the principal component analysis followed by linear discriminant analysis (PCA-LDA). Our study consists of testing both approaches on simulated and experimental Raman data. Within the simulated data, we investigated two scenarios: one with and one without background contribution. For both scenarios, we constructed 63 different simulations by changing the mean signal-to-noise ratio (SNR) and the shift in the peak position that was used to construct the abnormal class. A 10-fold cross-validation (10-fold CV) was used to evaluate the model performance in both scenarios. We could show that the functional approach (FPCA-LDA) performed better in region b of both Fig. 3 and Fig. 4, where the SNR and the shift in the peak position values are inversely proportional. However, both methods perform statistically similar in region a and region c of the same figures. In these both regions, either the quality of the spectra is low (low SNR) in combination with a small peak shift (region a) that both models do not work or the spectral quality is so good (region c) that both methods perform perfectly. These outcomes were similar for both scenarios (simulated Raman data with and without background). Then, we evaluated both approaches on experimental Raman spectra. Therefore, raw and pre-processed Raman data were used. Two cross-validation methods were implemented, the leave one batch out cross-validation (LOBOCV) and 10-fold CV. A slight improvement in the classification performance is shown when comparing the mean sensitivities between the PCA-LDA and the FPCA-LDA methods for the raw and the pre-processed experimental data. Moreover, these results were similar regarding the use of the cross-validation methods. This outcome of analyzing functional data on the experimental data is in accordance with the simulation data with or without background since the signal-to-noise ratio in the experimental data is high. However, the functional approach is sensitive to the choice of the number of basis functions, making this selection a very challenging task, which is an advantage of the classical approach. Furthermore, in terms of computation power, the functional version takes more time since an additional approximation step is needed before applying the functional PCA.

In conclusion, the functional data analysis can be considered a promising tool for analyzing Raman spectra, especially when the quality of the data is low. This property makes functional data analysis a great tool to analyze spectra which were acquired fast, or in vivo which both yield low-quality Raman spectra. It might be that the function data analysis is not so sensitive to a missing wavenumber calibration, because functions are utilized, and that functional data analysis can be used with spectra with a different spectral resolution. These points need further investigation and will be investigated in the future.

## Supplementary information

ESM 1 (DOCX 1010 kb)
