# Supplementary material for: Comparison of functional and discrete data analysis regimes for Raman spectra
Source: Anal Bioanal Chem. 2021 May 15;413(22):5633–44. doi: 10.1007/s00216-021-03360-1 (PMC8410698; doi:10.1007/s00216-021-03360-1)
Supplement: Supplementary file 1 — (DOCX 1010 kb) [file 216_2021_3360_MOESM1_ESM.docx]

**Supplementary Information**


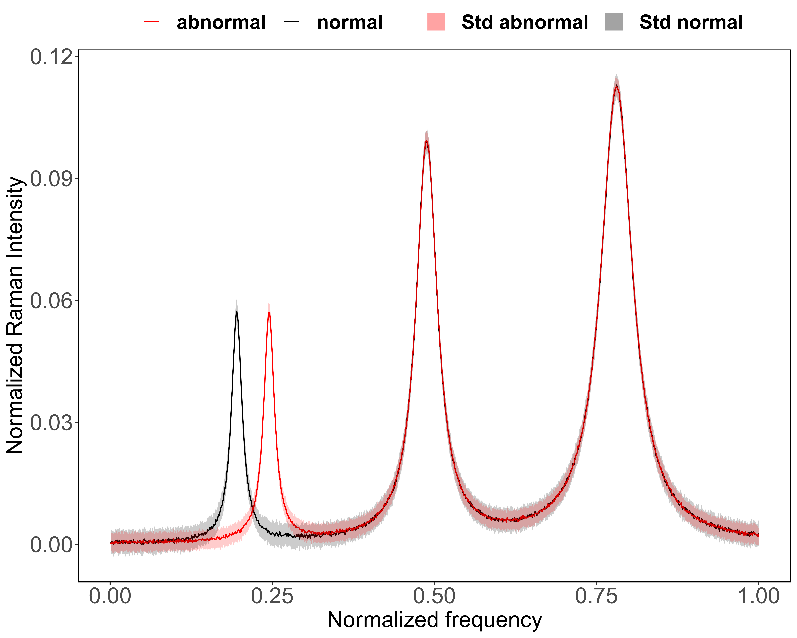


**Fig. S1** The mean spectra for the simulated Raman without background per class in the case of $\Delta\tilde{\upsilon}=0.05$ and $SNR=30$. The mean spectra and its standard deviation for the normal simulated Raman are shown in black. While the mean spectra for the abnormal simulated Raman and its standard deviation are shown in red.

**
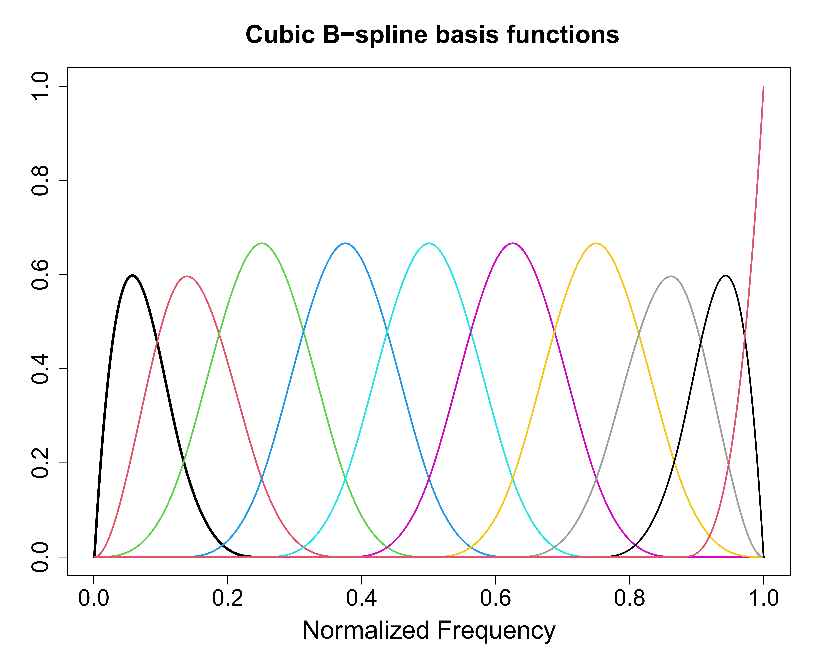
**

**Fig. S2** Cubic B-spline basis functions used in the FDA approximation. An illustration of the first ten basis functions is shown.


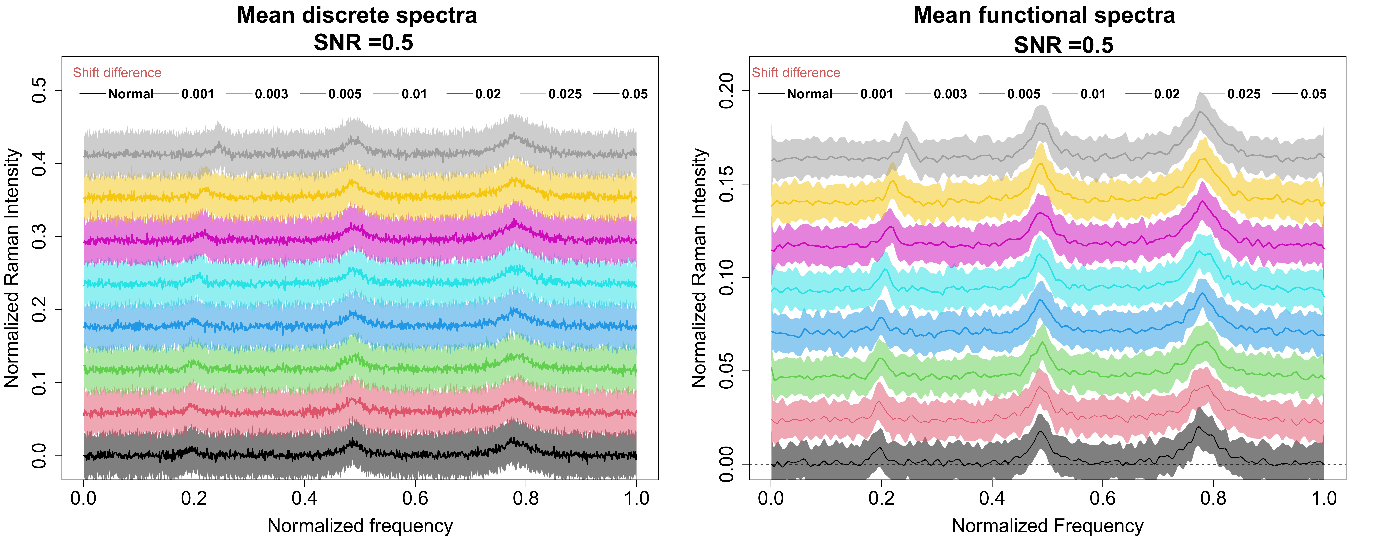


**Fig. S3** The discrete and the functional mean spectra per each shift for $SNR=0.5$for the simulated Raman without background. The left plot represents the discrete simulated Raman mean spectra, where the black plot represents the normal class, and the colored spectra represent the abnormal class for each $\Delta\tilde{\upsilon}$. On the right plot, the functional approximation using 190 basis functions is illustrated. Reduction of noise is shown in addition to the improvement in the shape of the peaks.


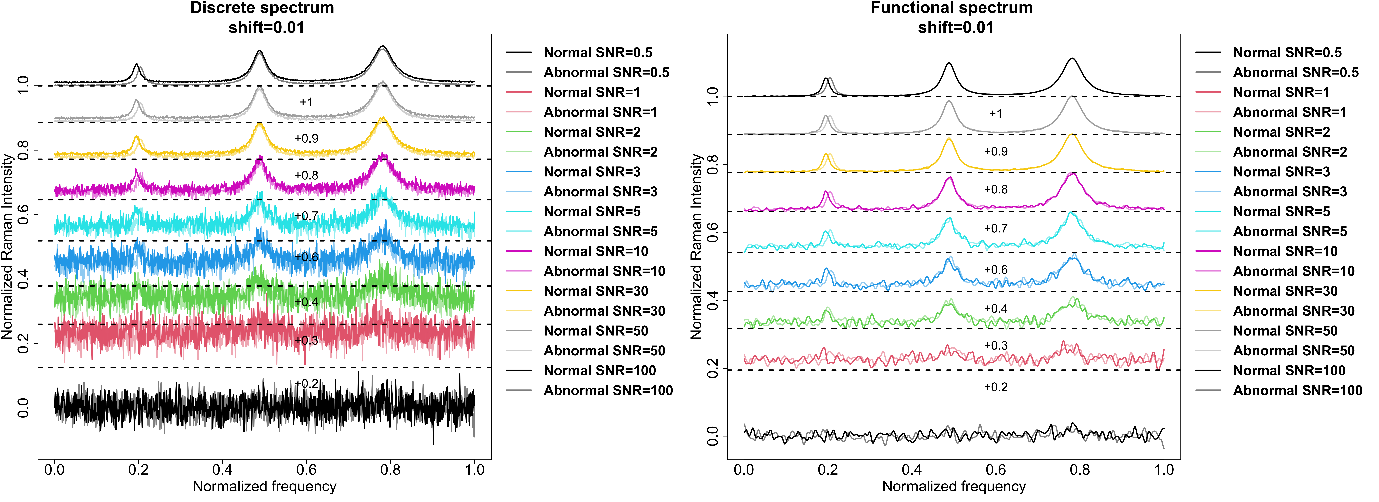


**Fig. S4** The discrete and the functional spectra per each SNR for $\Delta\tilde{\upsilon}=0.01$within the simulated Raman without background. The left plot represents on each row a simulated Raman spectrum for the normal and abnormal class. While the right plot represents the functional approximation of these spectra using 190 basis functions. Reduction of noise is shown in the right panel.


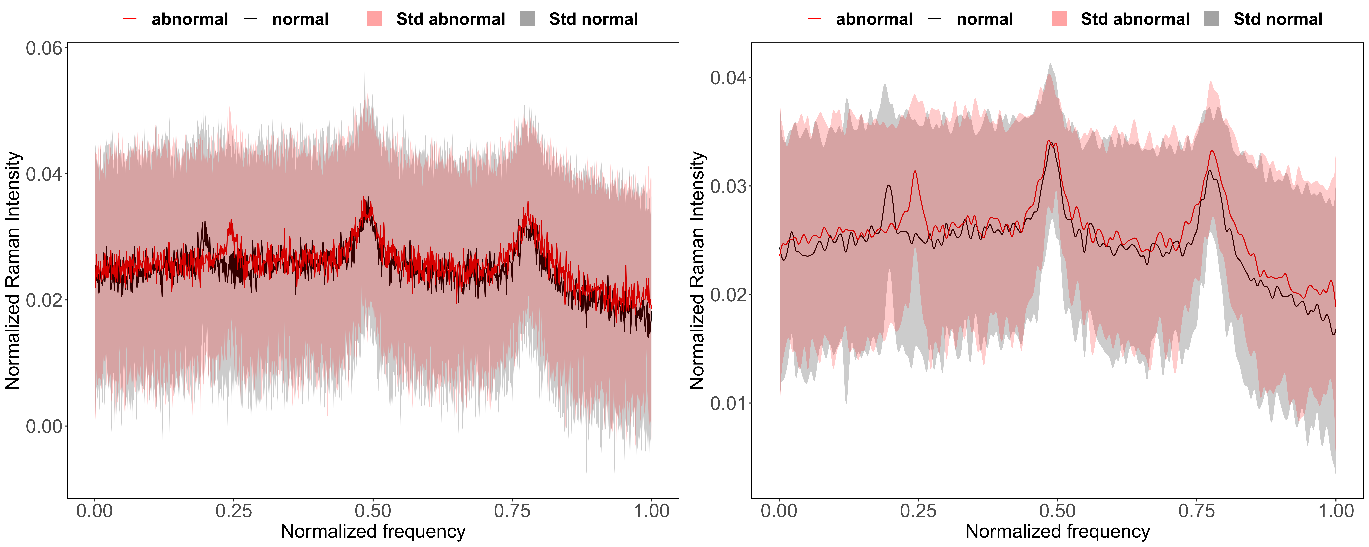


**Fig. S5** The mean spectra per class for the simulation with background ($\Delta\tilde{\upsilon}=0.05$ and $SNR =0.5$). The left plot represents the discrete simulated Raman mean spectra per class. While the right plot represents the functional version using 180 basis functions of these simulated Raman spectra.


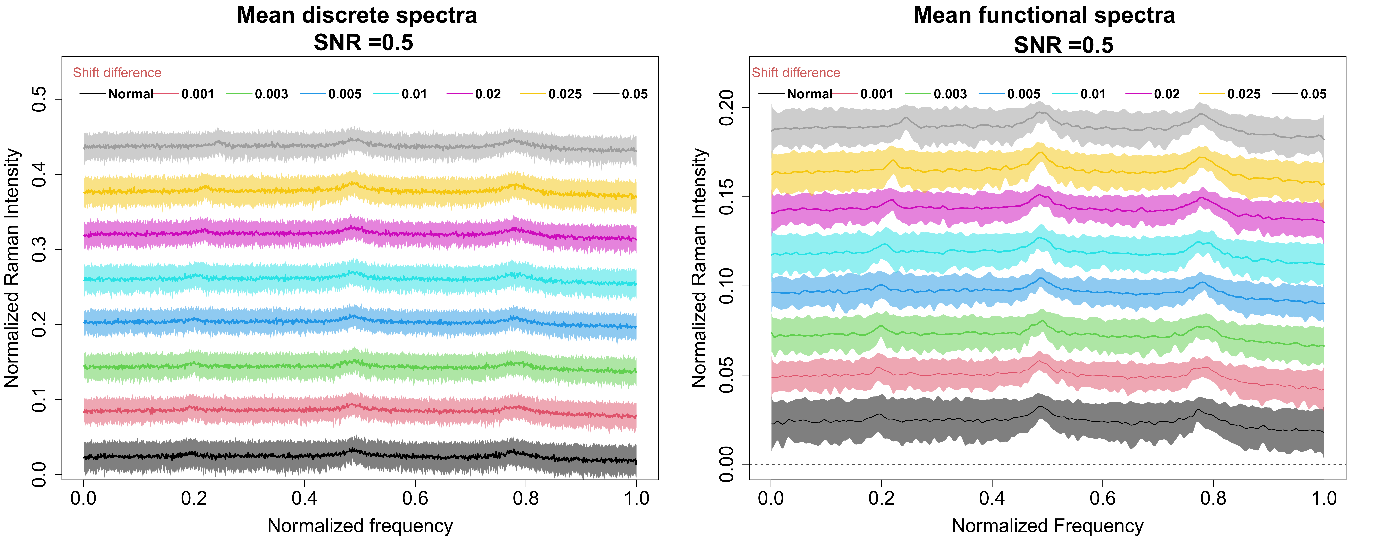


**Fig. S6** The discrete and the functional mean spectra per shift for $SNR=0.5$in the scenario with background. The left plot represents the discrete simulated Raman mean spectra. The black spectrum represents the normal class, while the colored spectra represent the abnormal class specified by $\Delta\tilde{\upsilon}$. While the right plot represents the functional approximation using 180 basis functions. Reduction of noise is shown in addition to the improvement in the peaks shape.


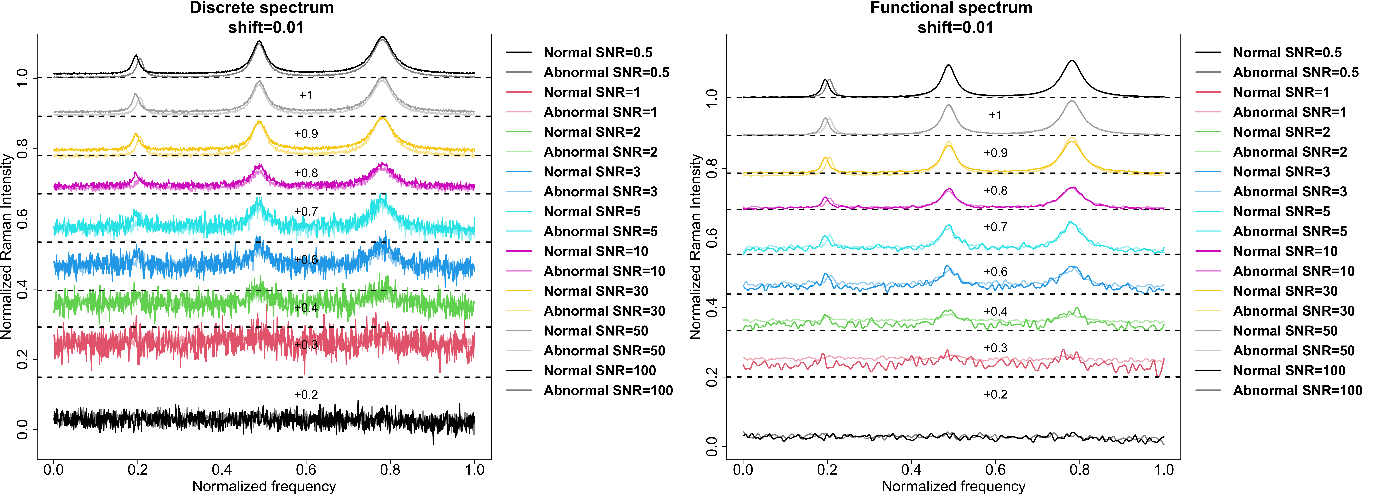


**Fig. S7** The discrete and the functional simulated Raman spectra per SNR for $\Delta\tilde{\upsilon}=0.01$with background. The left plot represents in each row a simulated Raman spectrum for the normal and abnormal class. While the right plot represents the functional approximation of these spectra using 180 basis functions. Reduction of noise is shown in the right panel.


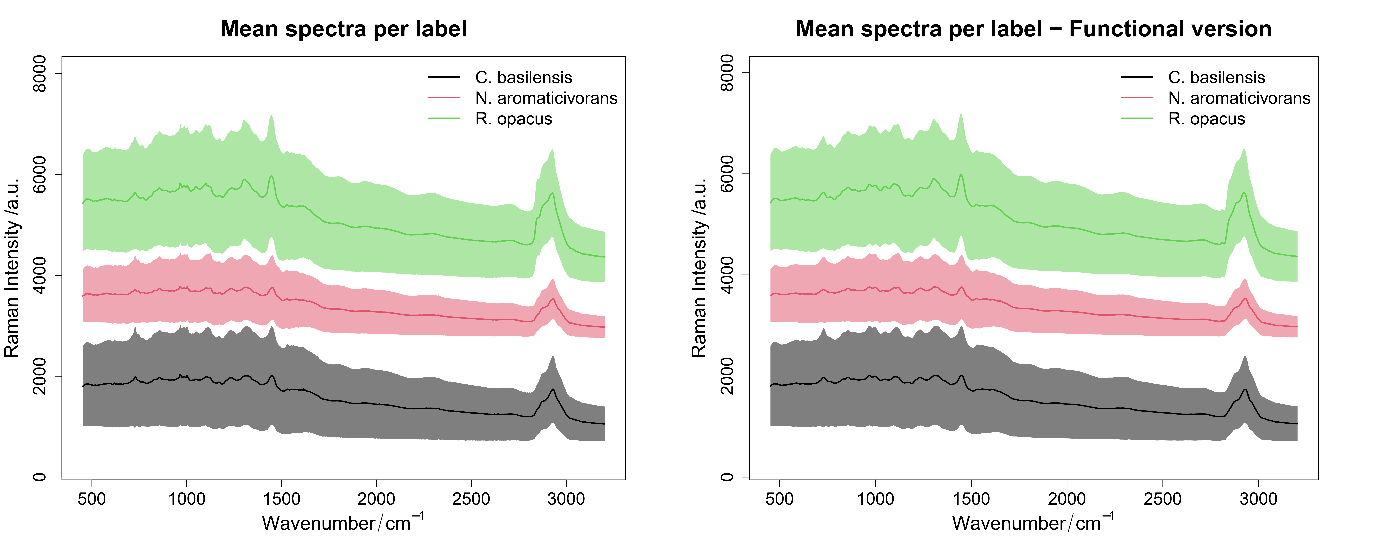


**Fig. S8** The mean spectra per label for the raw experimental data and their functional approximation. The mean discrete spectra per each label are shown on the left, while their functional mean spectra are illustrated on the right.


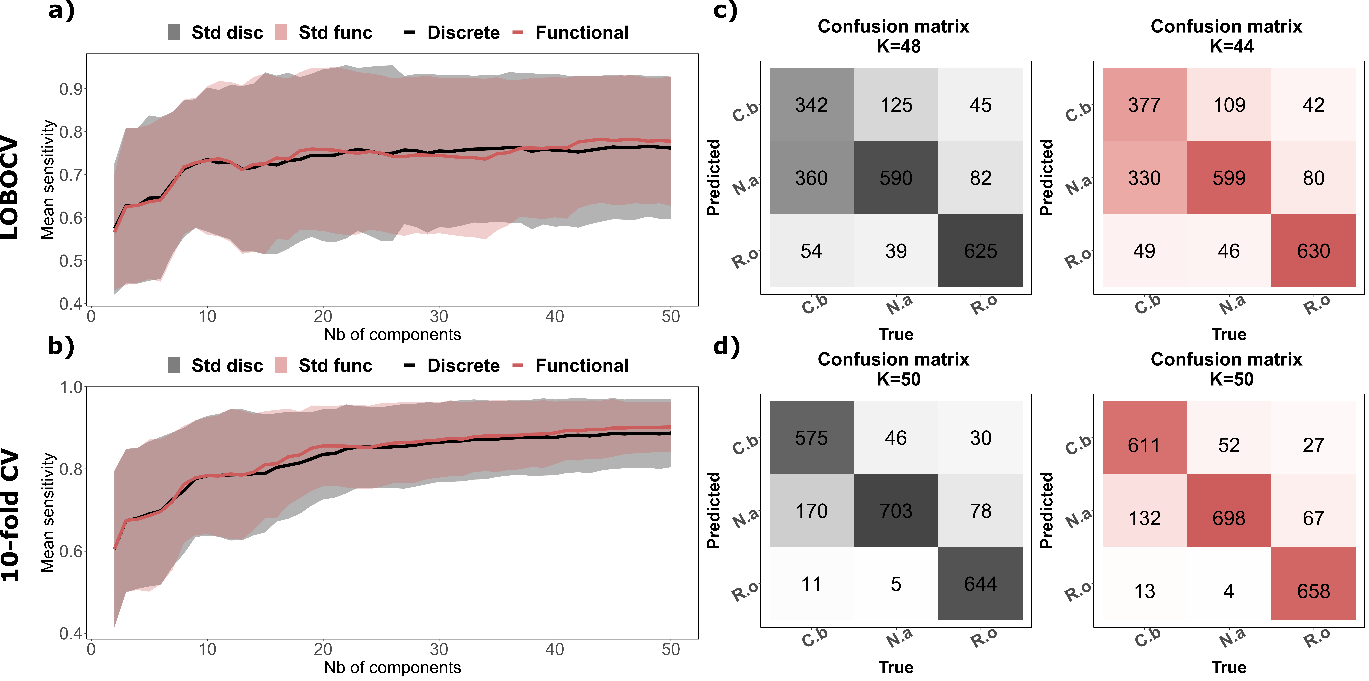


**Fig. S9** The mean sensitivity and the confusion matrices of PCA-LDA and FPCA-LDA methods using LOBOCV and 10-fold CV on the raw Raman data. Panel a) represents the mean sensitivities of the PCA-LDA and the FPCA-LDA methods using LOBOCV in black and red, respectively. The confusion matrices of these models for the corresponding highest mean sensitivity are illustrated in c). Panel b) refers to the mean sensitivities of PCA-LDA and FPCA-LDA methods using 10-fold CV in black and red, respectively. The confusion matrices of the models for the corresponding highest mean sensitivity are illustrated in panel d).


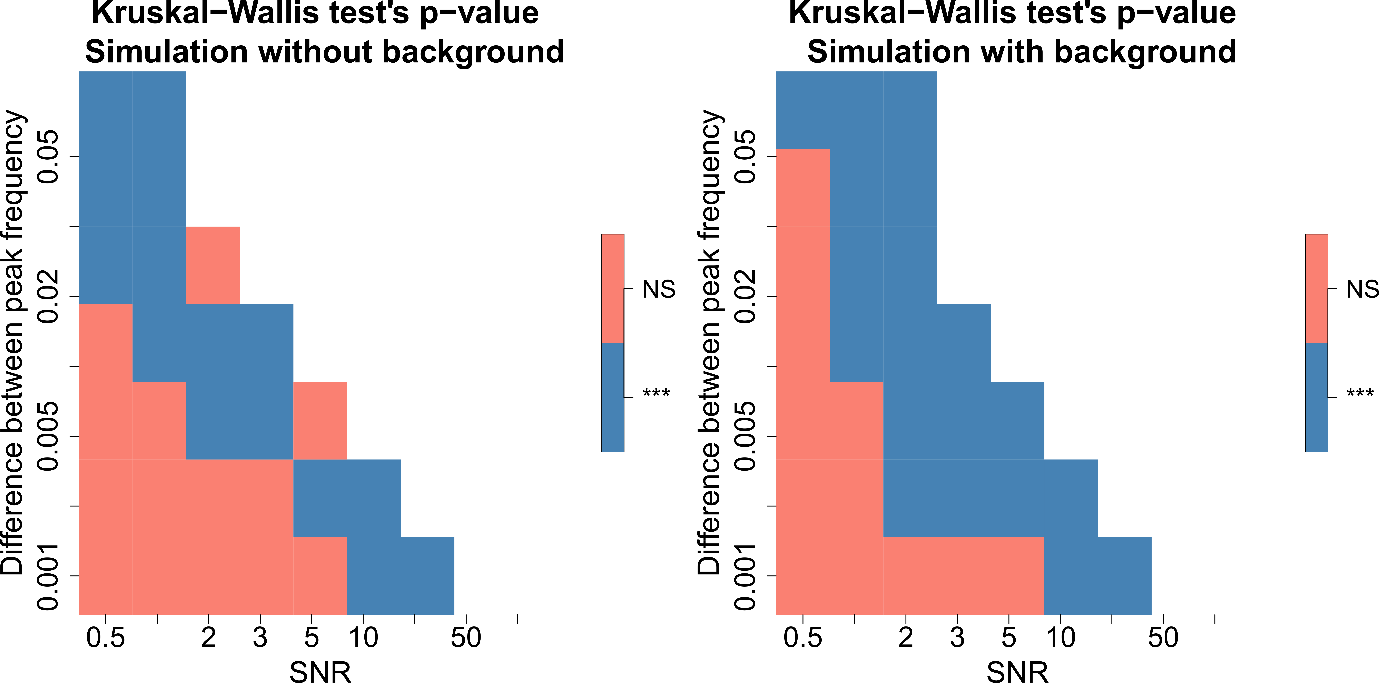


**Fig. S10** The p-values of the Kruskal-Wallis test for both simulation without and with background. The significance difference between PCA-LDA and FPCA-LDA classification is illustrated in blue while non significance difference is highlighted in red.

**Table S1** The parameter details of the simulated Raman spectra with and without background

| Parameters | Values |
| --- | --- |
| SNR | $\left\{ 0.5, 1, 2,3,5,10,30,50,100 \right\}$ |
| Labels | abnormal, normal |
| Shift peaks position $\Delta\tilde{\nu}$ used to construct the abnormal label | $\left\{ 0.001, 0.003, 0.005, 0.01, 0.02, 0.025, 0.05 \right\}$ |
| Number of pixels | 1024 |
| Peaks details: number, width, amplitude, position | 3, $\left\{ 0.01,0.02,0.03 \right\}$, $\left\{ 0.005,0.0175,0.03 \right\}$, $\left\{ 200,500,800 \right\}$ |
| Basis functions | Cubic B-spline basis of order 4 |
| $K$ | 190 for the simulated Raman spectra without background  180 for the simulated Raman spectra with background |

**Table S2** Mean intensity and standard deviation of specific wavenumber for the Pre-processed Raman spectra and its functional version displayed per microorganisms

| Wavenumber  / cm^-1^ | Mean intensity $\pm$ standard deviation | | | | | |
| --- | --- | --- | --- | --- | --- | --- |
|  | *R. opacus* | | *N. aromaticivorans* | | *C. basilensis* | |
|  | Pre-processed Raman data | Functional version | Pre-processed Raman data | Functional version | Pre-processed Raman data | Functional version |
| 954 | $131.92\pm81.84$ | $131.44\pm80.2$ | $113.89\pm62.84$ | $114.28\pm61.24$ | $212.74\pm83.42$ | $216.17\pm82.26$ |
| 1402 | $122.58\pm86.43$ | $121.72\pm85.33$ | $99.24\pm48.08$ | $97.97\pm45.84$ | $204.92\pm83.65$ | $207.33\pm82.02$ |
| 2933 | $603.64\pm307.98$ | $597.1\pm303.76$ | $501.82\pm214.03$ | $495.06\pm208.42$ | $1104.44\pm400.38$ | $1080.6\pm390.26$ |
